# Editorial: Preventing and treating liver diseases: medicinal and food plants, their metabolites as potential options

**DOI:** 10.3389/fphar.2025.1577547

**Published:** 2025-03-18

**Authors:** Rongrui Wei, Wenmin Liu, Chunsu Yuan, Chunlei Zhang, Zhipei Sang, Qinge Ma

**Affiliations:** ^1^ College of Medicine and Health Science & School of Life Science and Technology, Wuhan Polytechnic University, Wuhan, China; ^2^ College of Chemistry and Pharmaceutical Engineering, Nanyang Normal University, Nanchang, China; ^3^ Tang Center for Herbal Medicine Research, Department of Anesthesia and Critical Care, Committee On Clinical Pharmacology and Pharmacogenomics, Pritzker School of Medicine, The University of Chicago, Chicago, IL, United States; ^4^ School of Traditional Chinese Pharmacy, China Pharmaceutical University, Nanjing, China; ^5^ School of Pharmaceutical Sciences, Hainan University, Haikou, China

**Keywords:** hepatoprotective, bioactive natural products, pharmacological target, molecular mechanisms, liver disease

Liver disease is seriously endangering human health in the world. It has become the leading cause of death in the word (Israelsen et al., 2024). The presence and progression of liver fibrosis led by hepatic inflammation is the main predictor of liver-related death across the entire spectrum of steatotic liver diseases (Taru et al., 2024). A combination of recent advancements of widely available biomarkers for early detection of liver fibrosis together with considerable advancements in therapeutic interventions offer the possibility to reduce morbidity and mortality in patients with liver disease (Dabrowska et al., 2024). Thus, it is necessary to discover new hepatoprotective drugs to prevent and treat liver diseases. The medicinal and food plants have unique advantages in terms of safety and compliance, which have always been an important source for finding new hepatoprotective drugs (Xu et al., 2024). Therefore, medicinal and edible plants are the important sources for researching and developing new hepatoprotective drugs, which has the advantages of safety, multi-targets, and multi-pathways. We are honored to serve as topic editor of Frontiers in Pharmacology, and compile “*preventing and treating liver diseases: medicinal and food plants, their metabolites as potential options*”.

The study by Han et al. discussed about the anti-cholestatic mechanism of zhuyu pill on rat model, which was induced by naphthyl isothiocyanate. In this study, some important methods were used to evaluate anti-cholestatic activity, including histopathology, serum biochemistry, mRNA-Seq, and qRT-PCR. As a result, zhuyu pill could obviously enhance the indexes of biochemical blood and liver histopathology of rats, regulate lipid metabolism pathway, and alleviate symptom of inflammation, and so on. Some important genes were verified by qRT-PCR, including Acox2, Cyp2a1, Cyp1a2, Cyp2c11, and Ephx2. In a word, zhuyu pill displays significant anti-cholestatic effect, which indicates that zhuyu pill is a promising drug to treat cholestasis in the future.

Li et al. made a review on bioactivities of Rutaecarpine derivatives by consulting related references. Rutaecarpine is an important alkaloid, belonging to pentacyclic indolopyridoquinazolinone type, which is isolated from *Evodia rutaecarpa* for the first time. Rutaecarpine possesses multiple therapeutic functions in clinical practice. In this review, the structures of Rutaecarpine derivatives were modified to seek better properties and potency. This review summarized diverse bioactivities and mechanisms of Rutaecarpine derivatives, including anti-tumor, anti-inflammatory, anti-Alzheimer, anti-fungal, anti-atherogenic activities*.* These compounds inhibited acetylcholine, cyclooxygenase-2, phosphodiesterase 4B, phosphodiesterase 5, and topoisomerases (Li et al., 2023).

Nawaz et al. made a review on approaches for treating chronic hepatitis C. In this review, the treatment progress of hepatitis C virus was summarized and assessed. A comparison was made between traditional interferon/ribavirin treatment and herbal methods rooted in traditional medicine. This review made an updated summary of diverse hepatitis C virus genome, along with pathogenesis, natural variations, and the impacts of economic, social, dietary, environmental factors. In summary, this review summarized the complexity of the hepatitis C virus genome and explored the potential and advantages of medicinal plants to treat hepatitis C virus infection.

Feng et al. evaluated the hepatotoxic and hepatoprotective effects of matrine by meta-analysis. The radar charts and 3D maps were used to analysize the related data from multiple databases. It was found that matrine possessed bidirectional effects by measuring AST, TG, ALT, TC, MDA, CAT, TNF-α, and SOD levels. The effective dosage (10–69.1 mg/kg) of matrine for bidirectional effect was determined by three-dimensional analysis. Moreover, the high liver protection and low toxicity dosage (20–30 mg/kg/d) of matrine was summarized in this review. The molecular docking and multiple pathways of hepatotoxic and hepatoprotective effects of matrine were summarized and assessed in this paper.

Liu et al. evaluated hepatoprotective activities of total cucurbitacins from *H. pedunculosum* in this review. Cucurbitacin B (70.3%), isocucurbitacin B (26.1%), and cucurbitacin E (3.6%) were regarded as the main components of total cucurbitacins from *H. pedunculosum* by UHPLC-MS/MS and RP-HPLC. Total cucurbitacins could reverse CCl_4_-induced metabolic changes with a dose-dependent manner, and impact energy and phenylalaninet pathways. The LD_50_ value (36.21 mg/kg) and NOAEL value (15 mg/kg) of total cucurbitacins were assayed in this study, respectively. In summary, total cucurbitacins of *H. pedunculosum* are promising potential hepatoprotective drugs in the future.

Kim et al. assayed additive activities of herbal medicines to treat non-alcoholic fatty liver disease with meta-analysis. In this review, eight trials with 603 participants were contained in this study. As a result, it was found that ultrasoundbased liver steatosis of herbal medicine group displayed a significant improvement, and the aspartate transferase levels of herbal medicine group decreased. In a word, herbal medicines displayed additive activities on lifestyle modification to treat non-alcoholic fatty liver disease, it established an important research foundation for the treatment of non-alcoholic fatty liver disease.

Zhou et al. studied *F. suspensa* (Thunb.) Vahl for the treatment of inflammation-associated diseases, and evaluated the signaling pathways of inflammation. In this study, forest plots, risk-of-bias summaries, funnel plots, and flow diagrams were applied to analyze the related data according to references. As a result, it was found that *Forsythia suspensa* (Thunb.) Vahl could alleviate inflammatory cytokine levels and improve anti-oxidant enzyme superoxide dismutase. Therefore, it is suggested that *F. suspensa* (Thunb.) Vahl will be a good potential drug for treating inflammatory diseases (Zhou et al., 2024).

Chang et al. evaluated the mechanism of Xie Zhuo Tiao Zhi for ameliorating chronic alcohol-induced liver injury in this paper. In this study, biochemical parameters and examinations were applied to seek mechanism of Xie Zhuo Tiao Zhi to alleviate alcohol-induced liver dysfunction. As a result, hepatic oxidative stress was ameliorated and Nrf2/Keap1 expression was enhanced by Xie Zhuo Tiao Zhi. Some pro-inflammatory factors were rescued by Xie Zhuo Tiao Zhi. Thus, it is concluded Xie Zhuo Tiao Zhi will be a potential drug in treating chronic alcohol-induced liver injury diseases in the future.

Elshaer et al. found that *S. costus* root ethanolic extract could alleviate NaNO_2_-induced hepatorenal toxicity by means of regulating apoptosis, inflammation, and metabolism. The study displayed that the NaNO_2_-treated group improved the expressions of TNF-α and P53, and reduced expressions of IL-4 and BCL-2. It was found that *Saussurea costus* root ethanolic extract could alleviate the toxic effects of NaNO_2_ and improve liver function by assaying hematological parameters and modulating histopathological architecture. In a word, *S*. *costus* root ethanolic extract could alleviate NaNO_2_-induced hepatorenal toxicity, which will serve as a promising detoxifying additive in the future.

Mounanga et al. made a survey with 97 participants in this study, and analyzed data by One-way ANOVA and *t*-test. The survey mainly selected 63 plants, which were belonging to 35 families. The common symptoms summarized in this survey included cough, fever, cold, and fatigue. The study data emphasized that male subjects (31–44 years) had university education level. It was found that some plants displayed the highest UV, RI, and, RFC values, including *Alstonia congensis*, *Annickia chlorantha*, *Carica papaya,* and *Zingiber officinale*. Therefore, this survey revealed that traditional medicines could alleviate COVID-19 symptoms, which suggested some traditional medicines were useful for preventing coronavirus infections.

Chu et al. assayed therapeutic activities on NASH with salidroside and clarified mechanism on C57BL/6J mice with methionine- and choline-deficient diet. The study revealed that salidroside possessed preventive and therapeutic effects for NASH *in vivo*, including alleviating inflammation, downregulating apoptosis, upregulating autophagy, and rebalancing immunity. It was found that salidroside might exert its multiple therapeutic effects by activating PPAR*α*. In a word, this research exhibited that salidroside possessed anti-NASH effect by regulating autophagy, apoptosis, and immunity, and alleviating inflammation via activating PPAR pathway.

Chen et al. Established C57BL/6J mouse model of NASH by feeding high-fat diet for 12 weeks, which was used to reveal mechanism of *P. cyrtonema* ethanol extract against non-alcoholic steatohepatitis. In this study, 211 metabolites were identified by UHPLC-MS/MS. The study showed *Polygonatum cyrtonema* ethanol extract could improve hepatocellular degeneration and steatosis. *Polygonatum cyrtonema* ethanol extract also could restore the expressions of SREBP1, AMPK, PPAR-*α*, SIRT1, and regulate and upstream molecules and canonical pathways by analyzing RNA-seq data. Thus, *P. cyrtonema* ethanol extract could alleviate NASH and activate AMPK/SIRT1 pathway to prevent and treat the non-alcoholic fatty liver disease.

Jiang et al. performed a systematic review to assay hepatoprotective and hepatotoxic effects with 2,3,5,4′-tetrahydroxystilbene-2-O-*β*-D-glucoside. In this review, 24 studies encompassing 564 rodents were selected and analyzed. It was found that 2,3,5,4′-tetrahydroxystilbene-2-O-*β*-D-glucoside showed certain bidirectional activity by measuring ALT and AST levels. Moreover, some important biochemical indicators had been tested to evaluate hepatoprotective and hepatotoxic effects, including MDA, TNF-α, TC, TG, IL-6, SOD, and IFN-*γ*. It was found that the toxic dosage (51.93–76.07 mg/kg/d) and the protective dosage (27.27–38.81 mg/kg/d) were summarized in this review. Furthermore, 2,3,5,4′-tetrahydroxystilbene-2-O-*β*-D-glucoside could generate bidirectional effects on liver injury by PPAR, NF-κB, JAK/STAT, TGF-*β*, PI3K/Akt pathways.
